# The Eyes as a Mirror of the Unconscious: What Pupil Dilation Reveals About Unconscious Analytic Thought in Insight Problem Solving

**DOI:** 10.3390/jintelligence14090211

**Published:** 2026-09-02

**Authors:** Laura Macchi, Manuela Turco, Daniele Inglese, Laura Caravona

**Affiliations:** Department of Psychology, University of Milano-Bicocca, 20126 Milano, Italy; m.turco12@campus.unimib.it (M.T.); d.inglese1@campus.unimib.it (D.I.); laura.caravona@unimib.it (L.C.)

**Keywords:** insight problem solving, unconscious analytic thought, pupillary dilation, eye tracker, dual process theories

## Abstract

Recent studies suggest forms of unconscious processing in classical insight problem solving. In particular, the *unconscious analytic thought* (UAT) approach speculates that the creative act of restructuring such very demanding problems implies a form of high-level unconscious thought, mainly active during incubation. The present study aims to investigate whether pupillary dilation, typically associated with conscious cognitive effort, can also represent an indicator of unconscious cognitive effort (UAT). In the experimental condition, participants attempted to solve the problem and, during the incubation phase, performed a series of single-digit arithmetic calculations. In the control condition, participants only performed the arithmetic calculations. Results showed a significant interaction between group (solvers, non-solvers and control) and temporal course on pupillary dilation. In particular, solvers, conversely to the other two groups, showed high and constant pupillary dilation during all the incubation phase, suggesting the maintenance of constant cognitive effort at an unconscious layer not attributable to anything other than the processing of the insight problem solution. In the other two groups, instead, the pupil size—starting at the same level as solvers—progressively decreased during the execution of the arithmetic task. Our results are discussed in the light of the debate on dual process theories.

## 1. Introduction

Since the seventies, in an attempt to explain the coexistence of judgment biases and accurate reasoning, dualistic theories of thought have played a crucial role. They hypothesise the existence of two distinct modes of information processing, characterised by different mechanisms, times and resources. The first, automatic, fast, and intuitive, allows to efficiently tackle familiar and everyday tasks without requiring a great deal of cognitive effort (System 1). The second, on the other hand, slower, reflective and deliberative, comes into play when greater cognitive effort is needed, such as in new, complex or uncertain situations (System 2; Evans & Frankish, 2009; Kahneman, 2011). This view of thought has been challenged by cognitive phenomena that show a more complex and dynamic functioning of the mind, as in the case of insight problems, whose solution does not seem to be fully explained by either intuitive thinking or reflective thinking (Macchi & Bagassi, 2015; Mosconi, 1990, 2016).

### 1.1. Insight Problem Solving

Insight problems and procedural problems differ both in the nature of their difficulty and in the solution processes. Procedural problems are usually resolved through a gradual and sequential process (step-by-step), just like the person in a labyrinth. In these problems, the difficulty is related to the calculation, to the number of operations required and to the amount of information that must be processed and retained in memory (e.g., the “Cryptoarithmetic problem” by Simon & Newell, 1971). According to the established tradition that started with Simon and Newell (1971) and has continued through to the present day (Weisberg, 2018), a problem solver makes a selective search within the problem space, without an impasse and restructuring, via procedural conscious thinking. This theoretical tradition claims that conscious analytic thought gathers information from the failure to search for a new strategy. However, this approach does not explain how the solution is discovered from merely observing that the attempt has failed.

In insight problems, on the contrary, the difficulty does not appear to depend on the complexity or onerosity of the processing to be carried out, nor in the ability to evaluate the suitable strategy consciously. The issue consists in the difficulty of changing the representation, which may not be addressed by working memory capacity (DeCaro et al., 2016; Gilhooly & Fioratou, 2009) or conscious retrieval from memory of solutions or crucial parts of solutions to reproduce. In these cases, in which the complexity of the calculations does not play a significant role in the difficulty of the problem, a more appropriate abstract model than the labyrinth would be that of the *misunderstanding* (Macchi & Bagassi, 2015). It necessarily relies on a non-conscious, implicit processing (i.e., incubation) and therefore represents a challenge to the dualistic current theories of thought, regarding the central role of consciousness, as, in these cases, restructuring (i.e., the change in representation) is not performed consciously (Macchi, 2026; Mosconi, 1990, 2016). Incubation is in fact a necessary condition for reaching the solution, but not sufficient. It allows the process but does not guarantee success; however, when it is inhibited, for example, by requiring participants to simultaneously verbalize the solution process, resolution will be impeded. The study of the verbalization effect offered some possibilities to investigate the thought processes underlying insight problem solving (Macchi & Bagassi, 2012; Macchi et al., 2025). Furthermore, Caravona and Macchi (2023) explored the effects of different types of incubation tasks (visual, numerical and verbal), with varying degrees of attentional focus and cognitive effort (non-demanding, low-demanding, and high-demanding), on the resolution of insight problems. The most effective, regardless of its nature, emerged to be the low-demanding task, as, although requiring attentional focus, leaves resources available for the unconscious analytic restructuring process, resulting in a high percentage of success in solving the problem shortly after the incubation phase. Overall findings support the hypothesis of *unconscious analytic thought* (UAT; Macchi & Bagassi, 2012; Bagassi & Macchi, 2016; Macchi, 2026), according to which the restructuring required in insight problem solving implies a covert thinking process that includes a relevant, analytic and goal-oriented search.

### 1.2. Special Process View and Business-as-Usual Approach

Insight problems have been a major focus of study in the psychology of thought; the mechanisms that enable their resolution have been investigated for decades, giving rise to models and theories developed from various perspectives, with the aim of explaining how the mind arrives at the so-called “Aha moment”.

The *business-as-usual approach* (Bowden et al., 2005; Chronicle et al., 2004; Chuderski & Jastrzębski, 2018) claims that insight does not derive from a sudden process, but from a gradual and deliberate analysis of the problem, conducted through conscious thinking strategies, logical rules and already known operational schemes. However, despite the robustness of this theoretical framework, the model fails to explain precisely how and when the change in the representation of the problem occurs. Failure or impasse, in fact, does not necessarily lead to restructuring, and the conditions that favor its emergence remain not clearly defined (Macchi, 2026).

Conversely, the *special process view* (Ball et al., 2015; DeCaro et al., 2016; Jung-Beeman et al., 2004; Kershaw & Ohlsson, 2004; Knoblich et al., 1999) supports the idea that qualitatively different processes come into play in insight problems than those involved in procedural problems. Various studies (Jung-Beeman et al., 2004; Öllinger et al., 2008) suggest that the resolution of such problems takes place largely through processes that operate outside conscious control. As for Gestalt psychologists, the existence of an incubation phase that precedes insight is recognised; however, some authors describe these processes as the result of automatic and associations by chance (Ash & Wiley, 2006; Fleck, 2008; Schooler et al., 1993).

However, traditionally, insight problem solving has been considered as evidence of giftedness (e.g., Sternberg & Davidson, 1986) and insight problems (e.g., the “Water Lilies problem”) are often used to assess reflective abilities (Cognitive Reflection Test—CRT, Frederick, 2005). According to Gestalt psychology, solving an insight problem represents an example of “productive thought”. In addition to the reproductive activities of thought, there are processes that create, “produce” what does not yet exist. It is characterised by a switch in direction that occurs together with the transformation of the problem or a change in the understanding of an essential relationship. Then, the solution to classical insight problems seems to involve specific high cognitive abilities, to interpret and disambiguate the task in the light of the context, questioning a purely associative explanation (Macchi & Bagassi, 2015; Macchi, 2026).

Recently, Salmon-Mordekovich and Leikin (2023) proposed to consider the two perspectives in an integrated way, postulating that they refer to two different thought processes, but both capable of enabling insight problem solving. According to the authors, however, analytic thought processes, consistent with the *business-as-usual* approach, would represent the standard method of resolution, while intuitive processes, described by the *special process approach*, would constitute a less frequent and exceptional method. Although this proposal represents an interesting attempt to overcome the theoretical opposition between the two approaches, it would seem to inherit both the limitations associated with them: on the one hand, as in the *business-as-usual approach*, it is still unclear how the restructuring of the problem actually takes place; on the other hand, as in the *special process approach*, the definition of the processes involved in the incubation phase and the subsequent emergence of the insight remains insufficient.

### 1.3. The Interpretative Function and the Emergence of Unconscious Analytic Thought

Our view of thinking considers context processing, which is always relevance-oriented, as cutting across both intuitive and analytic, reflexive thinking (Macchi, 2026). Context, “situations” as Barsalou (2012) would say, appears to enhance cognitive processing by increasing the “breadth and specificity of inference”. Rather than randomly searching through every piece of knowledge stored in long-term memory, the system selectively draws on information and skills relevant to the situation at hand. Reasoning, therefore, entails constructing specialised representations and the relations among them in support of goal pursuit. In classical reasoning tasks, however, communicative heuristics that incorporate contextual cues, the intention of the speaker and the aim of the task have usually been considered impediments to correct response (e.g., Stanovich & West, 2000).

From our perspective, this interpretative processing is instead the core of thinking, a way of functioning shared by both language and thought (Bagassi & Macchi, 2016; Macchi, 2026; Mosconi, 1990), conceived as a goal-oriented tendency to seek relevant connections between information. Linguistic mechanisms are deeply interpenetrated by interactive thinking, and, vice versa, communicative heuristics deeply inhabit our minds (Levinson, 1995).

According to this view, the restructuring process could be understood as a form of *re*-interpretation that includes both implicit and explicit processing. Restructuring cannot be fully supported by explicit thought, which is occupied by the fixation and by the default interpretation, or *Einstellung.* The impasse, the difficulty of reaching the solution, will activate an increasingly focused, implicit search resulting in a *re*-interpretation of the available data in light of the request and vice versa.

In light of these considerations, this approach identifies insight problem solving as a privileged domain of application, interpreting it as a process closely intertwined with the communicative dimension. From this perspective, problems are not simple logical structures to be solved, but communicative configurations whose understanding depends on the interpretation of information and the implicit intentions conveyed by it.

Indeed, in our view, the way of thinking involved in insight problem solving closely resembles the process involved in understanding an utterance, when a misunderstanding arises. In both cases, resolving the misunderstanding (“impasse”) requires moving beyond the default interpretation (“fixation”) and selecting a new meaning (“restructuring”) that better fits the context and the speaker’s intention. A new conception of unconscious, implicit thought informed by relevance emerges (Macchi, 2026; Macchi & Bagassi, 2012). As mentioned above, rather than adopting an abstract and decontextualised approach, the interpretative function enhances specific elements of the context, drawing on relevant information to support understanding. When meanings and relationships are familiar, the process is quick and immediate; by contrast, when novel or unusual interpretations are required, the most common and habitual ones prove ineffective, prompting a cognitive exploration, both conscious and unconscious, of new connections in search of a different configuration that better fits the problem (Macchi & Bagassi, 2015).

Our approach considers that the creative act of restructuring implies high-level implicit thought, a sort of *unconscious analytic thought* (Macchi, 2026; Macchi & Bagassi, 2012, 2015) informed by relevance, where *analytic thought* is to be understood as the act of grasping the crucial characteristics of the problem structure and not a gradual, step-by-step simplification of its difficulties.

Restructuring requires viewing the same data from a different perspective and discovering new relations that were not apparent in the initial representation. The process does not rely on exhaustive searches or abstraction, but rather depends on identifying a relationship between the data that is most pertinent to the problem’s aim. In this way, each individual element acquires a different meaning with respect to the others and to the whole, leading to a new representation of the problem and, ultimately, to its understanding, giving rise to a new gestalt.

In other words, to solve an insight problem the interrelations between the elements of the default interpretation have to be loosened in order to perceive new possibilities, to grasp, among many salient cues, the most pertinent information to the aim of the problem. This kind of process cuts across both conscious and unconscious thought.

Some theoretical models consider the link between explicit and implicit information processing (e.g., Goal + Associative Network Interaction—GANI, Gilhooly, 2016a; Explicit–Implicit Interaction, Hélie & Sun, 2010), as a continuous fluctuation between attention directed toward the task and temporary withdrawal into an internal train of thought (Stimulus-Independent Thought—SIT) thereby allowing alternative goals beyond those that caused the impasse to be explored. This internal processing that takes place during incubation has a neural correlate in the Default Mode Network (DMN; Raichle et al., 2001), a neural network that is active even in the absence of external stimulation and that continues to operate when the individual moves away from the problem. Moreover, “the sets of major brain networks, and their decompositions into subnetworks, show close correspondence between the independent analyses of resting and activation brain dynamics” (Smith et al., 2009, p. 13040). It is interesting to note the close correspondence between the brain activity during task performance and that recorded during the resting state. This correspondence reinforces the idea that the brain may process the same task dynamically in different ways, at a conscious and non-conscious layer, as the substrate of a restless mind (Smallwood & Schooler, 2006; Smallwood et al., 2008).

### 1.4. The Incubation Phase in Insight Problem Solving

In light of our perspective, the incubation phase assumes a particularly important function in solving insight problems. A few studies in the field of creative problem solving have shown that a pause, following several ineffective attempts, can favor the spontaneous onset of new intuitions (e.g., Gilhooly et al., 2015; Schooler et al., 2011; Smith & Beda, 2024).

A seminal review conducted by Sio and Ormerod (2009) confirmed the existence of a positive incubation effect, while also highlighting the role of various moderating factors. Among these, the nature of the problem, the type of activity performed during the break, and the duration of incubation significantly influence the effectiveness of the problem solving process. In this direction, a particularly relevant contribution is provided by the study by Caravona and Macchi (2023), which compared different incubation tasks characterised by different levels of cognitive load. The most effective task was the one that, although requiring attentional focus, left resources available for the unconscious analytic restructuring process during incubation to resolve the insight problem, resulting in a high rate of problem solving success shortly after incubation. These results support the idea that the restructuring required in insight problem solving involves a covert thinking process that includes a relevant, analytic, and goal-oriented search.

### 1.5. From Pupil to Mind: Pupillary Dilation in Psychology

Despite the growing attention to the role of unconscious analytic thinking in insight problem solving, one question remains unanswered: how can we observe its activity if it occurs below the threshold of consciousness? Given that individuals have no conscious access to the activity of unconscious analytic thinking, it is particularly challenging for researchers to investigate its functioning.

To overcome this challenge, it is necessary to rely on objective indicators that can provide clues about any cognitive activity that occurs during the incubation phase. The use of physiological measures could represent a promising approach for indirectly observing unconscious processes, thereby offering a window into forms of mental processing that, by their very nature, elude introspection and subjective reports. In particular, psychophysiology has explored in depth the relationships between pupillary dilation and cognitive processes. It is well known, in fact, that changes in pupil size can be caused not only by sensory stimuli but also by thoughts, emotions, and cognitive loads (Mathôt, 2018).

Among the most studied cognitive processes in relation to pupillary dilation is cognitive effort: since the pioneering experiments by Hess and Polt (1964), it has been demonstrated that an increase in task difficulty corresponds to a proportional increase in pupillary dilation. The study by Kahneman and Beatty (1966) showed that pupil size reflects the cognitive load associated with working memory processes. Participants were given memory tasks of varying difficulty (digit recall, word recall, and digit transformation), while changes in pupil size were recorded during the different phases of the task. Specifically, in the digit transformation task, subjects were read a sequence of digits (e.g., 5-2-9-1) and asked to transform it by adding “1” or “3” to each digit (e.g., 6-3-0-2). The task made the mental effort progressively more demanding as the sequence grew longer, as the number to be added grew larger. Kahneman observed that pupil dilation corresponded exactly to the intensity of the cognitive effort: the more complex the transformation, the more the pupils dilated.

This raises the central question of our research: if conscious cognitive effort leads to an increase in pupil size, is it possible that unconscious cognitive effort, such as that hypothesised to characterise the incubation phase in insight problem solving by the UAT, might also produce a similar physiological response?

Laeng et al. (2012), for example, claim that pupil size can serve as a window onto the preconscious, indirectly revealing mnemonic activity not yet accessible to conscious awareness. An experiment conducted by Laeng et al. (2007) on amnesic patients showed that, despite the absence of explicit memory, the pupil responded differently to familiar and unfamiliar stimuli, suggesting implicit information processing.

In the field of visual perception, Tamietto et al. (2009) ran a study on patients with unilateral destruction of the visual cortex and revealed that emotional facial expressions, presented within the blind visual field, were still able to elicit appropriate emotional responses in patients and that their pupillary responses showed greater dilation in reaction to the emotional expressions presented.

Finally, another study that specifically investigated pupillary dilation during the resolution of Compound Remote Associates (CRA) tasks is that of Salvi et al. (2020), in which the authors analysed changes in pupil size. Participants were asked to indicate the moment of their response and to classify it as insight or analytic (procedural). The results showed that, in cases of insight solutions, pupil size increases approximately 550 ms before the response, peaking around 200 ms prior to the response. This effect was not observed in solutions classified as analytic. The pupillary increase was also detected in both correct and incorrect responses, suggesting that it reflects the emergence of intuition rather than the accuracy of the solution.

In light of this evidence, the present study aims to explore whether pupillary dilation may serve as a physiological indicator of unconscious analytic thought (UAT) that according to our hypothesis should occur during the incubation phase of insight problem solving.

### 1.6. Research Hypothesis

Given the difficulty of classic insight problems, we hypothesise that the activity of unconscious analytic thinking, which is activated during the incubation phase in individuals who have previously encountered an insight problem, involves greater cognitive effort. Although this effort is not accessible at the conscious level, it is expected to be reflected in changes in the pupillary response.

From this perspective, it is expected that the UAT activity will be particularly evident in participants who successfully solve the problem, as they may have benefited from more effective unconscious processing during the incubation phase. Consequently, it is plausible to hypothesize that problem solvers will exhibit a distinct pattern of pupillary dilation during the incubation task compared to both non-solvers and a control group that performs the same task without prior exposure to the insight problem. More specifically, solvers are expected to maintain relatively high and stable levels of pupillary dilation over time, suggesting sustained unconscious cognitive processing of the problem, whereas non-solvers and control participants are expected to show a gradual reduction in pupil size, reflecting primarily adaptation to the ongoing task.

## 2. Methods

### 2.1. Participants

The sample consisted of *N* = 91 participants. Eight participants were excluded because extensive signal loss prevented reliable estimation of pupil size throughout the recording, while 1 participant was excluded because medication use was considered likely to affect pupillary responses. The final sample therefore consisted of 82 participants.

The participants were aged between 19 and 47 years (*M* = 23.2; *SD* = 3.85). Of these, 55 were women (67%), 25 were men (30.5%), and 2 were non-binary individuals (2.5%). The participants were students at the University of Milano-Bicocca.

Recruitment took place through various channels: the SonaSystem university platform, flyers distributed on campus, word of mouth, and sharing the initiative on social media. Students who enrolled through SonaSystem received compensation in the form of university academic credits (CFU).

Regarding educational background, participants held a high school diploma or a bachelor’s and/or master’s degree. All participants signed an informed consent form, including authorization for the processing of personal data.

The study was approved by the Research Evaluation Committee of the Department of Psychology (CRIP) (RM-2025-946).

### 2.2. Measures

An EyeLink 1000 Plus eye tracker (SR Research Ltd., Ottawa, ON, Canada) was used to record pupil size; it was positioned below the monitor on which the stimuli were presented. Recording was performed in monocular mode on the right eye, with a sampling rate of 1000 Hz. The data were subsequently resampled to 100 Hz to facilitate file management and processing. To ensure accurate and consistent recording across participants, a chin rest was used to maintain a constant distance of approximately 60 cm between the participant and the monitor. The eye tracker was calibrated using a standard five-point procedure prior to the start of the experiment.

The monitor used to present the stimuli was an Acer model hn274h (595 mm wide and 338 mm high, with a resolution of 1920 × 1080 pixels and a refresh rate of 60 Hz). All stimuli were presented using E-Prime 3, version 3.0.3.219 (Psychology Software Tools, Pittsburgh, PA, USA).

Regarding the experimental material, the insight problem used in the experimental condition is Wylie’s “Three Boxes problem” (Wylie, 1957; Mosconi, 1990). The text of the problem is as follows:

“*There are three identical boxes. One contains two white balls, one contains two black balls, one contains one white ball and one black ball. Each box has a label on it. At first, each label matched the contents; then they have all been rearranged. The goal is to determine the contents of the three boxes by extracting as few balls as possible.*”^1^

This is a particularly complex problem: in Mosconi’s study, only 15% of participants were able to solve it. The key to solving it lies in the information provided by the labels, which, once rearranged, no longer correspond to the contents of the boxes. If you take a ball out of the box labeled “black and white” and, for example, find a white ball, you can deduce that the box contains two white balls, since it cannot match the label. Consequently, the contents of the two remaining boxes can be deduced by process of elimination, again applying the principle that the label does not match the contents.

The difficulty of the problem lies precisely in the fact that this crucial piece of information tends to be neglected. The wording of the problem, in fact, directs attention toward the balls rather than the labels, leading solvers to overlook the key element. To confirm this, Mosconi reformulated the problem by emphasizing the labels from the beginning. In the new version:

“*There are three identical boxes. Each box has a label. One says ‘two white balls,’ one says ‘two black balls,’ and one says ‘one white ball and one black ball.’ At first, each label matched the contents; then they were all rearranged. The goal is to determine the contents of the three boxes by extracting as few balls as possible.*”

(Mosconi, 1990, p. 287)

With this reformulation, the resolution rate rose to 60%. In the current study, the original version of the problem was used. The problem was also selected for its compatibility with the pupillometric procedure, as it requires neither drawing nor writing, unlike other classic insight problems such as the “Study Window problem” (Macchi & Bagassi, 2014) or the “Square and Parallelogram problem” (Wertheimer, 1959), which could have compromised the quality of the pupillary recording. The text of the problem was presented in black on a grey background, using the “Consolas” font in size 36.

We used an arithmetic task, adapted from the study by Caravona and Macchi (2023), consisting of a series of simple calculations (single-digit addition and subtraction), in both the experimental (as incubation task) and control conditions. This task proved particularly effective in facilitating insight problem solving (e.g., “Pigs in a Pen problem”) with a correct response rate of 46.8%, compared to 14.6% in the control group. Although it requires constant attention, it is a repetitive and relatively mechanical activity that does not fully absorb cognitive resources, thus leaving room for unconscious analytic processing related to the insight problem previously presented to the participants in the experimental condition. The calculations were presented one at a time in the center of the screen, in black on a grey background, using the “Consolas” font in size 36. They were the same and in the same order, with additions and subtractions alternating.

At the end of the experiment, a brief questionnaire was administered to gather additional information. Specifically, the participants’ gender, age, and educational level were recorded. In the experimental condition only, the questionnaire included questions designed to assess familiarity with the insight problem, to exclude the possibility that participants already knew it. Finally, in both conditions, a question was asked regarding perceived difficulty of mathematical calculations, with the aim of obtaining a subjective measure of the cognitive effort required. The questionnaire was completed without recording pupil size.

### 2.3. Procedure

The experiment was conducted at the University of Milano-Bicocca, in the Eye Movements Laboratory, equipped with the necessary instrumentation to carry out a pupillometry study. The laboratory environment was quiet and free from visual distractions, with black walls and a window covered by heavy curtains to completely prevent the entry of natural light. Throughout the experimental sessions, the artificial lighting remained switched off, and the laboratory was kept completely dark except for the light emitted by the monitor.

Inside the laboratory, a workstation was arranged for the participant, consisting of a computer used for stimulus and task presentation and an eye tracker positioned below the monitor. The experimenter operated from a separate workstation equipped with a second computer connected to the recording system, allowing real-time visualization of the participant’s screen, monitoring of the correct presentation of stimuli, and managing of the entire experimental procedure.

Before the participant’s arrival, a random assignment was performed to allocate each participant either to the experimental condition or to the control condition. Upon arrival, participants received a brief introduction to the instruments used in the study, including a description of the workstation and the eye tracker, and were asked to sign an informed consent form. Participants were also provided with several instructions to ensure the proper execution of the procedure, including the request to silence their mobile phones (including vibration mode) and either keep them in their pockets, switch them off, or place them away from the workstation. This precaution was intended to prevent light variations that could interfere with pupil recordings, as well as to minimize distractions during tasks.

Following this initial phase, participants were seated at their workstation. To ensure a constant distance from the screen throughout the experiment, a chin rest positioned approximately 60 cm from the monitor was used. Before the experimental tasks began, the height of both the chair and the chin rest was adjusted to provide a stable and comfortable position.

Subsequently, the eye tracker was calibrated using a five-point calibration procedure. Before the experimental tasks started, the word “wait” was displayed at the center of a gray screen for 5000 ms. This phase allowed the recording of pupil size in a resting state, in the absence of any stimulation, and served as the baseline measure for subsequent analyses.

For participants assigned to the experimental condition, the Delayed Incubation paradigm (Gilhooly, 2016b; Segal, 2004) was adopted, following the procedure used by Caravona and Macchi (2023) in terms of both timing and task structure. During the preparation phase, which lasted four minutes, the “Three Boxes problem” was presented, with a duration previously validated as sufficient to induce a cognitive impasse. During this phase, participants were free to reflect on the problem, either by reading it aloud or silently. The phase ended either when the available time elapsed or when the participant solved the problem. Participants were informed that a time limit was in place but were not told about its exact duration, to prevent them from monitoring the elapsed time.

If participants proposed a solution, they were instructed to report it aloud without looking away from the screen and maintaining their position on the chin rest. In case of an incorrect answer, the experimenter responded with the standardised statement: “The answer is not correct. You may continue thinking about it for as long as you have time. I will let you know when the time is up.” Any requests for help or assistance were handled with the standard response: “I am sorry, but I cannot help you.” These responses were used consistently with all participants.

At the end of the preparation phase, a new eye tracker calibration was performed, followed by the incubation phase, which lasted eight minutes. During this phase, simple arithmetic tasks consisting of single-digit additions and subtractions, identical to those used by Caravona and Macchi (2023), were presented on the screen one at a time. Participants were instructed to respond aloud and solve as many calculations as possible. The transition to the next calculation was controlled by the experimenter to prevent participants from using the keyboard, as even small body movements may affect pupil size (Kelbsch et al., 2019). No feedback regarding response accuracy was provided.

After the incubation phase, participants entered the post-incubation phase, during which the insight problem was presented again. There was no time limit during this phase: the task ended only when the participant either solved the problem or decided to give up.

The instructions were provided at the beginning of each phase, without informing participants that they would be asked to solve the problem again after completing the arithmetic task. This precaution was intended to minimize deliberate and conscious reflection on the problem during the incubation period.

For participants assigned to the control condition, the arithmetic task was presented immediately after eye tracker calibration. This task was identical to the one used during the incubation phase of the experimental condition and had the same duration (eight minutes). The arithmetic tasks presented in the control condition were identical to those used in the experimental condition, both in content and presentation order. The order of presentation was kept constant across all participants and across both conditions. This procedure follows recommended practice in pupillometry research, where stimuli should be held constant across experimental conditions so that any observed differences can be attributed exclusively to participants’ cognitive processes (Mathôt & Vilotijević, 2023).

In the control group, the eight-minute period did not obviously constitute an incubation phase because it was not preceded by exposure to an insight problem. Rather, the control condition was designed to isolate the effect of the cognitive effort required by solving arithmetic tasks on pupil dilation. Thus, any differences in pupil dilation between the two conditions could be attributed to the possible unconscious processing of the insight problem in the experimental condition rather than to the cognitive effort required by the arithmetic tasks.

In summary, the experimental condition included three phases (preparation, incubation, and post-incubation), whereas the control condition included only the arithmetic task phase.

At the end of the experiment, all participants completed a brief questionnaire. For participants in the experimental condition who had not found the correct solution, the questionnaire was administered before revealing the correct answer. Finally, a brief debriefing session was conducted during which participants were informed about the aims of the study, the hypotheses, and the experimental conditions.

## 3. Results and Discussion

### 3.1. Preprocessing

Due to the large amount of data produced by the eye tracker, which recorded pupil size at a sampling rate of 1000 Hz, the data were downsampled to 100 Hz to facilitate data management and analysis. The latency of the pupillary response to visual stimuli is approximately 200 ms, while the first cognitive effects on pupil size emerge after about 500 ms. As a result, even lower sampling rates, such as 20 Hz (one measurement every 50 ms), are considered sufficient in cognitive pupillometry studies where the main interest is the comparison between experimental conditions (Mathôt & Vilotijević, 2023).

In pupillometric studies, a preliminary phase of data preprocessing is required, i.e., the transformation of the raw data acquired during the experiment into a format suitable for statistical analysis (Mathôt & Vilotijević, 2023). In this study, preprocessing was conducted on one file for each participant, containing all relevant variables: pupillary size measurements, start and end markers for each experimental phase, a timestamp for each measurement and indicators related to blinks and saccades.

Missing data, due to tracking losses or temporary malfunctions of the eye tracker, were encoded as missing values (NaN) and excluded from the analyses. Blinks and saccades were automatically detected by the EyeLink 1000 Plus software (version 4.48) and flagged in the raw data files. The exported event markers identified all samples considered invalid because they were associated with blink or saccade related events, including samples surrounding these events when flagged by the EyeLink software. All flagged samples were coded as missing values (NaN) and excluded from subsequent analyses. No additional manual exclusion windows before or after blinks or saccades were applied, and no interpolation was performed. As an additional quality control step, the processed data of each participant were visually inspected to verify that no implausible pupil values remained after the automatic preprocessing. No residual artifacts requiring further correction were identified.

A further element considered in the preprocessing phase is the baseline pupil size. In this study, the baseline was calculated for each participant and subsequently included in the analyses as a covariate, to control for interindividual differences in pupil size.

In this study, the baseline was calculated using a 500 ms window within the waiting phase that preceded the start of the task, in both the experimental and control conditions.

All preprocessing procedures and statistical analyses were conducted using the free Jamovi software, version 2.7 (The jamovi project (2025). *jamovi* (Version 2.7) [Computer Software]. Retrieved from https://www.jamovi.org (accessed on 15 April 2026)).

### 3.2. Preliminary Analyses

Among the participants assigned to the experimental condition, none solved the insight problem during the initial preparation phase. However, following the incubation phase, 27.5% of the participants (*n* = 11) found the correct solution.

For the analyses, participants were classified into three groups based on both their experimental condition and their problem solving performance. The first group consisted of participants assigned to the control condition (*n* = 42). Participants assigned to the experimental condition were further divided into two subgroups according to their performance in the post-incubation phase: those who correctly solved the insight problem (solvers, *n* = 11) and those who did not reach the correct solution (non-solvers, *n* = 29). All subsequent between-group analyses were conducted using these three groups.

Before proceeding with the statistical analyses related to the research hypothesis, it was necessary to verify that the groups did not differ significantly with respect to several variables potentially influencing pupillary dilation. This preliminary step allowed us to ensure that any observed differences in the results could be attributed with greater certainty to the experimental manipulation, and not to pre-existing individual differences between groups.

The first variable to be controlled was age, as it is known to significantly influence pupillary response latency (Mathôt & Vilotijević, 2023). The average age of the entire sample was *M* = 23.2, *SD* = 3.85. To check for differences between the groups, a one-way ANOVA was conducted. The Shapiro–Wilk test indicated a violation of the assumption of normality. However, given the size of the sample, parametric analyses were carried out. The results showed that the groups did not differ significantly in terms of age, *F*(2, 79) = 0.535, *p* = .588. This result suggests that age did not influence differences in pupil size between groups.

A further check concerned the number of arithmetic calculations carried out and the number of errors made during the arithmetic task. These analyses were conducted to ensure that the groups did not differ in their performance on the arithmetic calculations, so as to exclude the possibility that any differences in pupil size could be attributed to a greater effort associated with the calculations rather than to the experimental manipulation.

As for the number of calculations performed (non-solvers: *M* = 219, *SD* = 31.8; control: *M* = 229, *SD =* 42.9; solvers *M* = 211, *SD* = 35.6), the Shapiro–Wilk test indicated a violation of the assumption of normality. A one-way ANOVA showed no significant difference between groups, *F*(2, 79) = 1.16, *p* = .317. This result suggests that the number of arithmetic calculations performed during the eight minutes phase was equivalent across the three groups.

With regard to the number of errors made in the arithmetic calculations (non-solvers: *M* = 2.34, *SD* = 2.38; control *M* = 3.17, *SD* = 2.49; solvers *M* = 2.45, *SD* = 3.56), a comparison was made between the three groups. The Shapiro–Wilk test indicated a violation of the assumption of normality, but in this case as well parametric analyses were carried out. The one-way ANOVA showed no significant differences between groups *F*(2, 79) = 1.01, *p* = .378.

To complement the previous analyses, a subjective measure of the participants’ perceived exertion while performing the calculations was also considered. In the questionnaire administered at the end of the experiment, participants were asked the following question: “Did you find the arithmetic task difficult?” (Likert scale ranging from 1 = not at all difficult to 5 = very difficult). The entire sample reported on average a low level of perceived difficulty (*M* = 1.65, *SD* = 0.807). A one-way ANOVA was then performed which showed no significant differences between the groups *F*(2, 79) = 0.392, *p* = .677.

### 3.3. Main Analyses

To analyse the trajectories over time of pupil size during the execution of the arithmetic task, the data were aggregated by calculating, for each participant, the average of pupil size in 10 s intervals throughout the entire phase (eight minutes), both in the experimental and the control condition. For each participant, therefore, 48 measurements were obtained, representing the average pupil size every 10 s.

In particular, to investigate these trajectories during the arithmetic task and compare the groups of solvers (*n* = 11), non-solvers (*n* = 29) and control participants (*n* = 42), an Individual Growth Model was estimated, using the GAMLj3 Mixed Models module (v3.6.5) in jamovi (version 2.7). Pupil size was modeled as the dependent variable and the participant identifier (*id*) was included as a cluster variable. The model included fixed effects for linear and quadratic time, baseline pupil size, group (solvers, non-solvers, control) and the interaction between group and time. The group variable was dummy-coded, with the non-solver group (coded 0) serving as the reference category. To help model convergence, continuous independent variables (baseline pupil size and time) were standardised as z-scores. Regarding the random effects, a random intercept for participant identifier (*Var* = 185,731) and random slopes for time, both linear (*Var* = 5517) and quadratic (*Var* = 5639), were included, with an unstructured covariance matrix for the random effects. The model parameters were estimated using restricted maximum likelihood (REML). Degrees of freedom for fixed effects were estimated using the Satterthwaite approximation. The intraclass correlation coefficient (*ICC*) was 0.913. The model converged successfully using the bobyqa optimizer.

The model showed a good fit to the data, explaining 96.6% of the variance when both fixed and random effects were considered (*conditional R*^2^ = 0.966; *LRT χ*^2^(15) = 11,647.243, *p* < .001). Fixed effects alone explained 59.2% of the variance (*marginal R*^2^ = 0.592) and contributed significantly to the model, *LRT χ*^2^(9) = 135.057, *p* < .001.

Among the results related to the main effects -although less informative than the interaction effects for our research question-, there was a significant effect of time, both in its linear component, *β* = −53.66, 95% CI [−73.1, −34.2], *F*(1, 79.1) = 29.328, *p* < .001, and in its quadratic component, *β* = 46.86, 95% CI [−27.1, −66.6], *F*(1, 79.4) = 21.595, *p* < .001, indicating an overall change in pupil size during the execution of the arithmetic task.

There was a significant effect of baseline pupil size, *β* = 554.50, 95% CI [465.3, 643.7], *F*(1, 78) = 148.470, *p* < .001, showing that individual differences in initial levels of pupil size constituted a robust predictor of the values observed during the arithmetic task. This result confirms the importance of controlling for baseline pupil size in pupil size analyses.

**Figure 1:**
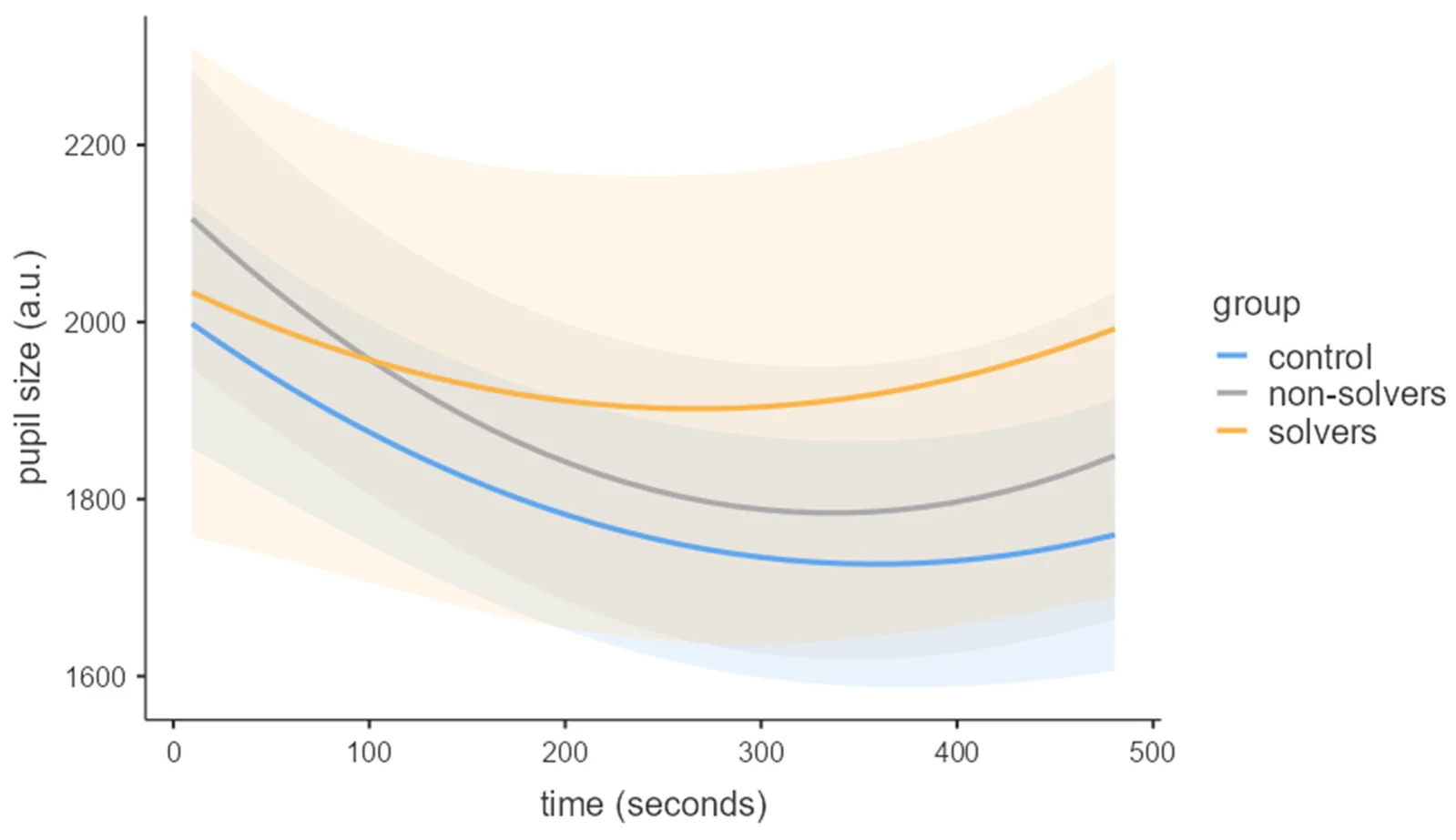
Pupil size trajectories during the execution of the arithmetic task in the solvers (*n* = 11), non-solvers (*n* = 29) and control (*n* = 42) groups. Time is expressed in seconds, whereas pupil size measurements are expressed in arbitrary units (a.u.) derived from the pixels detected by the eye tracking system’s camera. The effect of time on pupil size shown in the figure was estimated while controlling for baseline pupil size, which was included as covariate in the model. Shaded bands indicate 95% confidence intervals around the model-estimated trajectories.

There was no significant main effect of group, *F*(2, 75.5) = 0.527, *p* = .593, suggesting that the groups did not differ significantly in overall mean pupil size values, when these differences are considered independently of time trajectories. However, a significant interaction with linear time, *F*(2, 79) = 3.149, *p* = .048, highlights differences between groups in the trajectories that describe the linear time course of pupil size (Figure 1). Estimated marginal means indicated that, for the non-solver group, predicted pupil size decreased from 1949 (95% CI [1796, 2101]) at −1 SD of time to 1810 (95% CI [1650, 1970]) at the mean and to 1791 (95% CI [1622, 1960]) at +1 SD. The control group also showed a progressive reduction in estimated pupil size, from 1868 (95% CI [1741, 1996]) to 1755 (95% CI [1622, 1888]) and 1728 (95% CI [1587, 1869]). In contrast, the solver group showed relatively stable estimated values across time (1953, 95% CI [1702, 2204]; 1903, 95% CI [1641, 2165]; and 1929, 95% CI [1652, 2206], respectively). In particular, simple effects analyses revealed a significant effect of linear time for the group of non-solvers, *β* = −79.6, 95% CI [−108.1, −51.0], *F*(1, 79.1) = 30.737, *p* < .001, and for the control group, *β* = −70.8, 95% CI [−94.5, −47.1], *F*(1, 78.4) = 35.447, *p* < .001. In these two groups a statistically significant reduction in pupil size was observed over time: for each increase of 1 *SD* in the time variable, pupil size decreased by 79.6 units^2^ for the non-solver group and by 70.8 units for the control group. In contrast, no significant linear effect of time on pupil size was observed in the solver group, *β* = −12.7, 95% CI [−59.1, 33.8], *F*(1, 79.3) = 0.295, *p* = .589. This finding, namely the absence of a significant decrease in pupil size among participants who ultimately solved the insight problem, suggests that pupil size remained relatively stable throughout the incubation phase.

### 3.4. Discussion

Previous studies have shown that pupil size tends to increase when individuals are engaged in a cognitive task and, more specifically, that pupillary dilation increases as a function of task difficulty (Hess & Polt, 1964; Kahneman & Beatty, 1966). The present study investigated whether the activity of unconscious analytic thought, a form of high-level thinking operating below the threshold of awareness, could be detected through pupillary dilation. Two fundamental components of analytic thinking, according to Evans and Frankish (2009), are cognitive decoupling and hypothesis testing. In our view, cognitive decoupling may be understood as an advanced interpretative mechanism that allows individuals to disengage from an immediate or default interpretation and focus instead on the information most relevant to the current task. Several studies have linked unconscious analytic thought to insight problem solving (Macchi & Bagassi, 2012, 2015; Caravona & Macchi, 2023). This form of thinking is active during the incubation phase, when individuals are no longer consciously engaged in solving the problem but are occupied with a task that requires their conscious attention without exhausting all available cognitive resources (e.g., solving single-digit arithmetic tasks). The activation of this process allows the restructuring of the problem representation by revealing previously overlooked relationships and information that may lead to the solution (Caravona & Macchi, 2023). In the present study, we investigated whether pupillary dilation, generally considered an index of cognitive effort, could also serve as an indicator of analytic but unconscious thought when measured during the incubation phase. To this end, participants were divided into three groups—solvers, non-solvers, and a control group—allowing us to examine potential differences in the temporal dynamics of pupil size.

The results showed that participants who solved the insight problem exhibited a distinct pupillary pattern during the incubation phase compared to both non-solvers and the control group. Specifically, solvers showed a high initial level of pupil size that remained relatively stable throughout the entire incubation period, suggesting the maintenance of a sustained cognitive effort at an unconscious level. In contrast, in the non-solver group and control group, the large initial pupil size tended to progressively decrease during the same period. This difference does not appear to be attributable to the characteristics of the arithmetic task itself, as all participants performed the same task and showed comparable levels of performance. Preliminary analyses revealed no differences between groups in the number of arithmetic tasks completed, the number of errors made, or the perceived difficulty of the task.

The progressive reduction in pupil size observed during the arithmetic task among non-solvers and control participants may reflect an adaptation effect. The high level of pupil size observed at the beginning of the task likely indicates the greater effort required by a new activity, whereas the subsequent decrease may reflect the progressive automatization of arithmetic processing and, consequently, a reduction in cognitive effort (Mathôt, 2018). In the solver group, by contrast, the initial level of pupil size was comparable to that observed in the other two groups but did not show the subsequent decline over time. This sustained effort is consistent with the operation of an unconscious analytic process involved in the elaboration of the insight problem during the incubation phase.

Taken together, these findings suggest that the pupillary response observed in solvers reflects an additional source of cognitive effort beyond that required for performing the arithmetic task alone. From our perspective, during the incubation phase, solvers were engaged in the unconscious analytic processing of the previously presented insight problem. The maintenance of a stable level of cognitive effort may therefore reflect the activation of unconscious analytic thought, which continued to operate in parallel with the secondary task and subsequently facilitated the emergence of the solution during the post-incubation phase.

Furthermore, this interpretation is supported by evidence from the dual-task literature, which shows that simultaneous engagement in two consciously demanding activities is typically associated with performance decrements (Chen & Bailey, 2021). From this perspective, if participants had been consciously processing the insight problem during the incubation phase, a deterioration in arithmetic-task performance would have been expected. The absence of such differences therefore supports the hypothesis that the process observed in solvers developed outside of awareness.

Evidence pointing in the same direction comes from the influential study by Salvi et al. (2020), whose results show an increase in pupil size immediately preceding the solution of Compound Remote Associates (CRA) problems through insight. However, a subsequent reinterpretation proposed by Mansell et al. (2025) suggests that this effect may be partly attributable to emotional factors associated with the rapidity of the solution and the resulting subjective experience of surprise. According to this perspective, the greater pupillary size observed in insight solutions would primarily reflect an affective response associated with the rapid resolution of the task.

The results of the present study, however, are difficult to explain within this framework. Unlike previous studies, pupillary dilation was measured during the incubation phase, that is, while participants were exclusively engaged in solving arithmetic tasks and were not exposed to the insight problem itself. Under these conditions, it is implausible to attribute the observed pupillary response to emotional reactions associated with the discovery of the solution, as that event had not yet occurred.

A particularly relevant contribution to the interpretation of the present findings comes from the recent study by Stuyck et al. (2024), which investigated the involvement of cognitive resources in solving CRA problems through insight versus non-insight strategies by measuring vagally mediated heart rate variability (vmHRV), considered an indirect index of the engagement of prefrontal circuits associated with cognitive regulation.

In that study, vmHRV was interpreted as an index of the involvement of prefrontal circuits associated with working memory, a process that the authors explicitly linked to conscious access to mental representations. The results showed increased vmHRV during problem solving for both insight and non-insight solutions, suggesting, according to the authors, a comparable involvement of cognitive resources and conscious processes in the two solution modes.

The findings of the present study, however, seem to suggest a possible reinterpretation of the relationship between cognitive resource engagement and conscious awareness. Specifically, in the present study, solvers exhibited a stable level of pupillary size throughout the incubation phase, indicating the persistence of cognitive effort at an unconscious level across the entire eight-minute period, despite the absence of differences in arithmetic-task performance compared to non-solvers and participants in the control condition. This dissociation between physiological indicators and behavioral performance makes the hypothesis of intentional and conscious processing of the insight problem during the concurrent arithmetic task less plausible.

Considering both the present findings and the evidence reported by Stuyck et al. (2024), it would be interesting to further investigate the relationship between unconscious processing and neural activity during insight problem solving. Future research could integrate the experimental paradigm adopted in the present study with neurophysiological measures capable of monitoring the activity of brain regions involved during the incubation phase.

Indeed, several neuroscientific findings suggest that prefrontal circuits may also be involved in non-conscious cognitive processes. For example, several studies have reported the involvement of prefrontal networks in implicit forms of emotion regulation, including both automatic and controlled processes (Braunstein et al., 2017).

Moreover, the literature provides substantial evidence for the existence of complex cognitive processes operating outside conscious awareness. A meta-analysis by Gambarota et al. (2022), for instance, supports the existence of an unconscious visual working memory, showing that visual information presented outside awareness can nevertheless be used in goal-directed tasks. Similarly, cognitive control processes may also operate unconsciously. Lau and Passingham (2007) demonstrated that such processes can be associated with activation of the mid-dorsolateral prefrontal cortex, which is involved in inhibiting a competing task implicitly activated by a subliminal prime, thereby enabling the execution of the task required by the instructions.

Finally, some studies suggest the possibility that even higher-level cognitive processes, such as decision-making, can take place outside of awareness. For example, several studies reported by Dijksterhuis and Strick (2016; e.g., Bos et al., 2008) show, across a variety of decision tasks, evidence for true, active thought taking place unconsciously. Furthermore, even in the context of classical reasoning tasks, logical principles can be processed intuitively and without deliberation (De Neys & Pennycook, 2019).

The possibility that resource-demanding cognitive processes may operate in the absence of direct conscious access is consistent with the pattern observed in the present study, in which the maintenance of cognitive load during the incubation phase appears to be accompanied by the continuation of insight problem-related processing that remained phenomenologically inaccessible to participants.

An additional aspect worth considering concerns the nature of the insight tasks employed in recent research. Several studies that have questioned or attenuated the distinction between insight and non-insight problem solving (e.g., Mansell et al., 2025; Stuyck et al., 2024) have primarily relied on Compound Remote Associates (CRA) tasks, which require the identification of remote semantic associations between words. Although such tasks may indeed be accompanied by subjective experiences of insight, they differ in important characteristics from classical insight problems based on representational restructuring.

More specifically, CRA tasks appear to rely more heavily on associative and semantic processes, whereas classical insight problems, such as the one used in the present study, require the overcoming of implicit constraints and the reorganization of the problem’s mental representation. This distinction may have important theoretical implications, as it suggests that findings obtained using CRA tasks are not necessarily generalizable to all forms of insight problem solving.

From this perspective, the present findings appear particularly informative. The stable pattern of pupil size observed among participants who solved the problem following the incubation phase may reflect persistent processing specifically associated with insight problems that involve a substantial restructuring component. This interpretation may help explain why some recent studies have reported reduced differences between insight and non-insight problem solving. Such findings may depend, at least in part, on the type of task employed and on the cognitive processes predominantly involved.

More broadly, the present study may point toward moving beyond a strictly dichotomous conception of cognitive processes, characteristic of traditional dual-process theories. Throughout the entire eight-minute incubation period, solvers exhibited a stable level of pupil size, suggesting the persistence of resource-demanding cognitive activity over time. At the same time, the absence of differences in arithmetic-task performance relative to non-solvers and participants in the control condition makes it difficult to attribute this pattern to conscious and intentional processing of the insight problem during the concurrent mathematical task.

Taken together, these findings suggest the existence of cognitive processes that, despite operating outside conscious awareness, retain characteristics typically associated with conscious analytic processing, including the sustained engagement of cognitive activity over time and the recruitment of mental resources. From this perspective, unconscious processing may not be reducible to purely automatic, immediate, or associative activation, but may instead constitute a more complex, dynamic, and cognitively demanding form of processing.

A potential critical aspect concerning our interpretation of pupillary responses could be the limited specificity of the pupil as a physiological marker. Although pupil size is a sensitive physiological index of cognitive processing, it is not specific to unconscious analytic thought and may, in theory, also be influenced by other cognitive and physiological factors. Nevertheless, several aspects of the experimental design support our interpretation. During the arithmetic task phase, all participants performed the same arithmetic task, and the preliminary analyses revealed no significant group differences in arithmetic performance or perceived task difficulty. Furthermore, the distinction between solvers and non-solvers emerged only in the post-incubation period, whereas the pupillary trajectories analysed in the present study were recorded before the insight solution was produced. Although alternative explanations cannot be completely excluded, these methodological considerations reduce the likelihood that the observed group differences can be attributed to confounding factors.

The present findings are therefore consistent with more recent theoretical perspectives proposing the existence of an analytic form of unconscious thought and a more continuous, less rigidly dichotomous conception of cognitive functioning, according to which analytic and resource-demanding processes can operate in the absence of direct conscious access.

## Data Availability

The raw data supporting the conclusions of this article will be made available by the authors on request.

## Notes

1 Participants were presented with the Italian version of the problem. The English version reported in this article is provided for the reader’s convenience. The Italian text was as follows:
*“Ci sono tre scatole identiche. Una contiene due palline bianche*, *una contiene due palline nere*, *una contiene una pallina bianca e una nera. Su ogni scatola c’è un’etichetta. Dapprima ciascuna di esse corrisponde al contenuto; poi sono state tutte cambiate di posto. Si vuol conoscere il contenuto delle tre scatole tirando fuori il minor numero possibile di palline.”* (Mosconi, 1990, p. 284)

2 Pupil size measurements are reported in arbitrary units derived from the pixels detected by the eye tracking system’s camera.
